# Validity and repeatability of the EPIC physical activity questionnaire: a validation study using accelerometers as an objective measure

**DOI:** 10.1186/1479-5868-5-33

**Published:** 2008-06-02

**Authors:** Anne E Cust, Ben J Smith, Josephine Chau, Hidde P van der Ploeg, Christine M Friedenreich, Bruce K Armstrong, Adrian Bauman

**Affiliations:** 1 Centre for Physical Activity and Health, School of Public Health, University of Sydney, Sydney, Australia; 2 Centre for Molecular, Environmental, Genetic and Analytic Epidemiology, University of Melbourne, Melbourne, Australia; 3 Department of Health Sciences, Monash University, Melbourne, Australia; 4 Division of Population Health and Information, Alberta Cancer Board, Calgary, Alberta, Canada; 5 Sydney Cancer Centre, Royal Prince Alfred Hospital, and School of Public Health, University of Sydney, Sydney, Australia

## Abstract

**Background:**

A primary aim of the European Prospective Investigation into Cancer and Nutrition (EPIC) cohort study is to examine the association between total physical activity levels (comprising occupational, household and recreational activity) and the incidence of cancer. We examined the validity and long-term repeatability of total physical activity measurements estimated from the past-year recall EPIC questionnaire, using accelerometers as an objective reference measure.

**Methods:**

Participants included 100 men and 82 women aged 50–65 years. Criterion validity was assessed by comparing the physical activity estimates from the EPIC questionnaire with total activity estimated from the average of three separate 7-day accelerometer periods during the same (past-year) period. Long-term repeatability of the EPIC questionnaire was assessed by comparing the responses from the baseline and 10-month administrations. Past-year EPIC estimates were also compared with the Friedenreich Lifetime Total Physical Activity Questionnaire to examine whether recent activity reflected lifetime activity.

**Results:**

Accelerometer total metabolic equivalent (MET)-hours/week were positively associated with a total physical activity index (Spearman rank correlation ρ = 0.29, 95% confidence interval (CI) 0.15, 0.42) and with non-occupational activity estimated in MET-hours/week (ρ = 0.21, 95% CI 0.07, 0.35). Stratified analyses suggested stronger correlations for non-occupational activity for participants who were male, had a lower BMI, were younger, or were not full-time workers, although the differences in correlations between groups were not statistically significant. The weighted kappa coefficient for repeatability of the total physical activity index was 0.62 (95% CI 0.53, 0.71). Spearman correlations for repeatability of components of activity were 0.65 (95% CI 0.55, 0.72) for total non-occupational, 0.58 (95% CI 0.48, 0.67) for recreational and 0.73 (95% CI 0.66, 0.79) for household activity. When past-year activity was compared to lifetime estimates of activity, there was fair agreement for non-occupational (ρ = 0.26) activity, which was greater for household activity (ρ = 0.46) than for recreational activity (ρ = 0.21).

**Conclusion:**

Our findings suggest that the EPIC questionnaire has acceptable measurement characteristics for ranking participants according to their level of total physical activity. The questionnaire should be able to identify the presence or absence of reasonably strong aetiological associations when either recent or long-term activity is the responsible factor.

## Background

Physical activity is an important modifiable risk factor for several types of cancer, including colon and breast cancers, and possibly prostate, endometrial and lung cancers [1-4]. For practical reasons, most epidemiological studies use questionnaires rather than objective measures to document physical activity. However, physical activity is a complex and variable behaviour [5], and the ability of epidemiological studies to determine the relationship between physical activity and chronic diseases such as cancer is heavily dependent on the validity of their self-reported measures.

The European Prospective Investigation into Cancer and Nutrition (EPIC) on-going prospective cohort study was initiated primarily to examine the associations between diet, lifestyle and the incidence of cancer in over 500,000 participants in 10 western European countries. The EPIC questionnaire assesses past-year physical activity in occupational, recreational and household domains [6]; its validity has been examined in two previous studies. Pols and colleagues [7] reported correlations ranging from 0.26 to 0.81 when using three-day activity diaries as a reference measure, however this method has the potential for correlated measurement error [8]. Furthermore, in that study, the EPIC questions were interspersed with other physical activity questions in a longer instrument, which may have influenced the psychometric properties [7]. Wareham and colleagues [9] developed a four-level physical activity index, based on reported occupational, cycling and sports activity in the EPIC questionnaire, and found that it successfully ranked levels of activity and cardio-respiratory fitness as measured by heart rate monitoring and sub-maximum oxygen uptake. However, this index was not representative of 'total' physical activity because it excluded all light-moderate intensity activities (e.g. walking, do-it-yourself activities, gardening), which contributed 85% of participants' reported time in non-occupational activities, as these light-moderate activities were poorly correlated with the objective measures [9]. The insensitivity of heart rate monitoring to walking and lower-intensity activities is likely to have contributed to this finding [10,11]. Given that light and moderate intensity activities are the main contributors to total physical activity energy expenditure [12], especially in women [13], there is a need to examine whether total activity (including these light-moderate activities) is accurately measured by the EPIC instrument. Another important consideration is whether or not recent physical activity, as estimated by the EPIC questionnaire, can be used to infer aetiological associations with long-term physical activity, as long-term exposure is thought to be important in cancer aetiology.

As the EPIC study will continue to provide important results on the association of physical activity with risk of developing cancer and other chronic diseases, and these results will be incorporated into population-level physical activity guidelines, data from our validation study will enable better interpretation of EPIC study findings on total physical activity and cancer risk, and give guidance for use or adaptation of this questionnaire in other studies.

The objectives of this study were 1) to assess the criterion validity of total physical activity estimated from the EPIC questionnaire, using three 7-day accelerometer periods as an objective reference measure; 2) to evaluate the long-term repeatability of the EPIC questionnaire over 10 months; and 3) to compare agreement between the EPIC questionnaire and the Friedenreich Lifetime Total Physical Activity Questionnaire (LTPAQ) [14].

## Methods

### Study population

Eligible study participants included men and women aged 50–65 years living in Sydney, New South Wales (NSW), Australia. Volunteer participants were recruited between June and November 2005, from NSW state-wide Health Survey participants (63% of cohort), workplaces (33%), and by word-of-mouth (4%). Compared to the NSW general population, our study had a slightly higher proportion of participants who were male, or employed [15]. In our study 76% of men and 62% of women were overweight or obese compared to 70% and 57% respectively, in those of similar age in the NSW population [15].

Eligible participants were contacted initially by mail, with follow-up by mail and telephone. Of 401 people who were sent an information package, 189 (47%) gave consent and completed baseline data collection and 186 completed all aspects of the study including three accelerometer monitoring periods and the final questionnaire. After exclusion of four subjects with insufficient accelerometer data, 182 participants were included in the analysis.

### Study design

**Figure 1:**
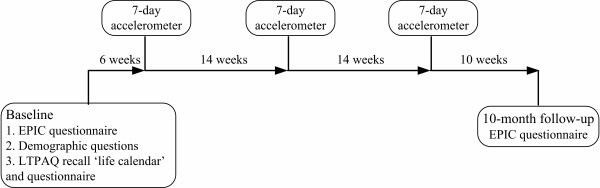
**Study design for the physical activity validation study**. Study design for the European Prospective Investigation into Cancer and Nutrition (EPIC) physical activity questionnaire validation and repeatability study, Sydney, Australia, 2005–2006.

Each participant completed the EPIC self-administered and LTPAQ telephone-administered physical activity questionnaires at baseline. During follow-up, subjects wore an Actigraph (MTI) accelerometer for three separate 7-day periods, each 14 weeks apart to capture seasonal variation. The EPIC questionnaire was completed again about 10 months after baseline (figure 1). All study materials were mailed to participants. The study was approved by the University of Sydney Human Ethics Committee and the NSW Department of Health Ethics Committee, and written informed consent was obtained from subjects.

### EPIC questionnaire

The EPIC questionnaire assesses past-year physical activity in occupational, leisure and household domains [6] [see Additional file 1]. For occupational activity, both current employment status and the level of physical activity carried out at work (non-worker, sedentary, standing, manual, heavy manual) were recorded. For recreational and household activities, participants reported the duration of activities during a typical week in the past year, in summer and winter. Household activities included housework, home repair, gardening and stair climbing. Recreational activities included walking, cycling and sports activities. Metabolic equivalent intensity values (METs), defined as the ratio of the metabolic rate during an activity to a standard resting metabolic rate of 1.0 (4.184 kJ)·kg^-1^·hour^-1 ^[16], were used to estimate the overall level of recreational and household activity in MET-hours/week. The assigned MET values (using the EPIC data manual guidelines) were 3.0 for walking and housework, 4.0 for gardening, 4.5 for home repair (do-it-yourself work), 6.0 for cycling and sports, and 8.0 for stair climbing, as used in EPIC analyses [17,18]. Time spent in vigorous non-occupational activity was measured in two ways: first, in a separate question about self-reported time in activities causing sweating or faster heartbeat, and second, using the sum of time spent in activities with MET values ≥ 6 (i.e. cycling, sports and stair climbing). Time spent in light-moderate non-occupational activity was estimated using the sum of time spent in activities with MET values < 6 (i.e. housework, walking, gardening, home repair).

The EPIC continuous variable estimates include household and recreational activity but not occupational activity because duration and frequency of occupational activity were not asked in the questionnaire. Thus, to examine total physical activity, the level of occupational activity was cross-tabulated with combined recreational and household activities (in sex-specific quartiles of MET-hours/week) to create a total physical activity index categorised as inactive, moderately inactive, moderately active, and active [see Additional file 2], as used in previous aetiological analyses within the EPIC cohort [17,18]. We also assessed the 'Cambridge' physical activity index based on occupational, cycling and sports activity, that is, generally more intense activities, developed by Wareham and colleagues [9] [see Additional file 3].

### Friedenreich Lifetime Total Physical Activity Questionnaire (LTPAQ)

The Friedenreich LTPAQ records at interview the recalled frequency (days/week, months/year), duration (time/day, number of years) and intensity of each of a respondent's physical activities in four different domains (occupational, recreational, household, transportation) over the entire lifetime [14]. It has been used to show associations between physical activity and cancer risk in epidemiological studies. Test-retest correlations ranging from 0.72 to 0.87 have been shown for the different domains of this questionnaire [14]. The questionnaire was developed using cognitive-based methods [14,19], and uses a self-completed recall calendar in which participants list their occupation, transport, sports and other events for each year of their life. A copy of the completed calendar was used by both the interviewer and the participant during the interview as a recall aid.

Data were processed according to the current LTPAQ protocol (available from C.M.F.). MET values [16] were assigned to each reported recreational and active transportation activity. For each occupational activity, participants reported up to five descriptions of their main work activity, from which an average MET value was estimated. Occupational activities with a mean MET value of ≤ 1.5, indicating sedentary activity, were excluded from activity estimates. For household activity, participants reported light, moderate and heavy activities separately, and assigned MET values of 2.5, 3.5 and 4.5, respectively. Total physical activity was estimated as the combined lifetime average of occupational, household, recreational and active transportation activities in MET-hours/week.

### Accelerometer measurement

Participants wore an Actigraph accelerometer (model 7164, LLC, Fort Walton Beach, Florida, USA) [20,21] on the right hip, attached to an elastic belt, for three separate 7-consecutive-day periods during follow-up. Participants were instructed to wear the accelerometer during waking hours except when in water.

The monitor was initialized as described by the manufacturer and data collected in 1-minute epochs. Activity counts above 18,000 counts/min were censored as probable artefact. Consecutive strings of zero-count epochs lasting 20 minutes or more were assumed to be periods of non-wear [22]. The average number of non-wear periods per day was 2.6 (SD 0.6), which includes the expected non-wear periods in the morning and evening. In the interpretation of our results, we assumed that non-wear time was sedentary activity. We excluded from the analysis days with fewer than 10 hours of registered monitor wear, and weeks that had fewer than four days of valid data. Using these criteria, we excluded four participants (2%) with less than two valid weeks of accelerometer data. Of the 182 participants included in the analysis, the average wear-time was 14.7 (SD 1.2) hours/day and the average number of valid days was 19.4 (out of a possible 21). For each accelerometer period, we calculated weekly estimates of activity by multiplying the average daily estimates of activity (derived from valid days) by seven.

The Swartz prediction equation and cut-points [23] were used to convert the accelerometer counts into estimated time spent in light activity (< 574 counts/min), moderate activity (574–4944 counts/min) and vigorous activity (= 4945 counts/min). The Swartz equation was chosen because it was derived from a broad range of mainly light-moderate intensity lifestyle-related activities, which account for a large proportion of total activity in this population age-group. We classified sedentary activity as < 100 counts/min, as used in other studies [24,25]. MET-hours of activity were estimated by multiplying the hours spent in light, moderate and vigorous activities by 2.5, 4.5 and 6.5 METs, respectively [16], and these were summed to estimate total MET-hours/week of activity.

### Statistical analysis

Total physical activity was the main measure of interest, measured primarily using the 'total physical activity' categorical index, and secondarily as non-occupational activity in MET-hours/week from the EPIC questionnaire. Criterion validity was assessed by comparing these physical activity measures, as estimated from the EPIC questionnaire at 10 months, with total activity (sum of light, moderate and vigorous) estimated from the average of three 7-day accelerometer measures. The long-term repeatability of the EPIC questionnaire was assessed by comparing total activity, and activity in different domains (e.g. household, recreational) and intensities (light-moderate, vigorous), between the baseline and 10-month administration. Recent and lifetime physical activity were compared using the baseline EPIC and LTPAQ questionnaires.

As the data were not normally distributed, we used nonparametric tests and presented data as medians and interquartile ranges. Spearman correlation coefficients (ρ) with 95% confidence intervals (CI; derived using Fisher's z transformation) and Bland-Altman plots with 95% limits of agreement [26] were calculated as the main measures of agreement between (and within) the instruments. We used the Z-statistic and associated p-value to test whether the correlation coefficients were significantly different between groups [27]. Repeatability for categorical variables was assessed using weighted kappa statistics [28], using default weights based on categories ordered as 1, 2, 3 etc. The analyses were stratified by gender, median body mass index (BMI; ≤ 27.2, >27.2 kg/m2), median age (<58, ≥ 58 years) and employment status (full-time, other), to determine whether the questionnaire may be more or less accurate in certain groups of people. All analyses were performed using SAS Statistical Software (version 9.1, SAS Institute, Cary, NC), and statistical significance was inferred at two-sided P < 0.05.

## Results

### Participant characteristics and physical activity levels

A total of 100 men and 82 women with a mean age of 57.2 years participated in this study. Baseline characteristics of participants are shown in table 1. The mean BMI was 27.7 kg/m^2 ^(range 18.6–61.7) and 70% of participants were classified as overweight or obese. More than half the participants were in full-time employment (men 77.0%, women 43.9%).

**Table 1:** Baseline characteristics of participants in the EPIC physical activity questionnaire validation and repeatability study (n = 182)

	Number^a^	%^a^
Male	100	55.0
Female	82	45.1
Age (years)		
50–54	67	36.8
55–59	57	31.3
60–65	58	31.9
BMI category (kg/m^2^)		
Normal weight (BMI 18.5–<25)	55	30.4
Overweight (BMI 25–<30)	81	44.8
Obese (BMI 30+)	45	24.9
Marital status		
Single/separated/divorced/widow	43	23.8
Married/defacto	138	76.2
Highest education level		
Some high school	21	11.7
Completed high school	18	10.0
Technical college/other	82	45.6
University	59	32.8
Employment status		
Not employed/retired	41	22.6
Part-time or casual	27	14.9
Full-time	113	62.4

EPIC, European Prospective Investigation into Cancer and Nutrition

^a ^Missing data (<1%) excluded from calculations

Physical activity levels as estimated by the different assessment methods are reported in table 2. The estimated median total hours/week spent in physical activity was 46.7 for accelerometer measurement and 33.4 for the LTPAQ questionnaire (*P*_diff _<0.0001). The estimated hours/week of non-occupational activity was 20.3 using the EPIC questionnaire compared with 12.2 for the LTPAQ (*P*_diff _<0.0001). Full-time workers and non-workers/casual-workers had similar estimates of total activity using accelerometers, but when the EPIC non-occupational activity estimates were stratified by employment status, the median hours/week were 17.1 for full-time workers and 29.2 for non-workers/casual-workers (*P*_diff _<0.0001), which gives an indication of the overall contribution of occupational activity to total activity in this sample. Light to moderate intensity activities were the most prevalent activities in our population, accounting for more than 99% of total activity based on the accelerometer data.

**Table 2:** Physical activity levels as estimated from accelerometer measurement and EPIC and LTPAQ self-reported questionnaires

Physical activity assessment	Median (25^th^-75^th ^centile)	
	
	Hours/week	Counts
*Accelerometer*^a^		
Total counts per day		347328 (278084–428769)
Average counts per minute		398 (316–483)
		
		MET-hours/week
		
Total activity	46.7 (39.8–53.6)	160.0 (135.3–183.6)
Light intensity	25.9 (21.1–29.4)	64.8 (52.8–73.5)
Moderate intensity	19.5 (16.3–23.7)	87.6 (73.4–106.5)
Vigorous intensity	0.2 (0.0–0.7)	1.1 (0.3–4.3)
Sedentary time^b^	121.1 (114.1–128.1)	
		
*EPIC questionnaire (10 months)*		
Total non-occupational^c^	20.3 (13.6–31.6)	73.5 (52.6–116.0)
Recreational	9.0 (5.5–14.0)	34.5 (19.5–54.0)
Household	10.1 (5.2–20.1)	37.7 (19.0–68.8)
Vigorous activity, self-rated^d^	2.0 (0.0–5.0)	18.0 (0.0–45.0)
Vigorous activity, MET-assigned^e^	2.0 (0.3–4.8)	12.4 (1.6–28.8)
Light-moderate activity^e^	17.5 (10.5–28.0)	59.8 (34.3–96.0)
		
*LTPAQ questionnaire*		
Total activity^f^	33.4 (26.8–41.3)	98.6 (72.6–123.3)
Non-occupational	12.2 (7.2–19.4)	46.2 (29.6–72.2)
Occupational^f^	17.1 (10.6–24.1)	40.1 (23.2–58.5)
Recreational	3.0 (1.6–4.2)	15.5 (8.2–23.8)
Household	7.2 (3.5–13.7)	22.6 (11.6–45.9)
Active transportation	0.9 (0.5–1.4)	2.9 (1.7–4.5)
Vigorous activity, MET-assigned^e^	0.5 (0.1–1.5)	3.8 (0.7–12.4)
Light-moderate activity^e^	30.5 (23.6–38.9)	86.9 (65.6–112.8)

EPIC, European Prospective Investigation into Cancer and Nutrition; LTPAQ, Lifetime Total Physical Activity Questionnaire

^a ^Data are averages of three 7-day accelerometer periods. The Swartz cut-points [23] were used to estimate the time spent in light, moderate and vigorous intensity activity.

^b ^Sedentary time defined as <100 counts/min, and includes non-wear and sleep time; corresponds to 17.3 (16.3–18.3) hours/day

^c ^Combined recreational and household physical activity

^d ^Estimated from a question about time in activities causing sweating or faster heartbeat

^e ^MET-assigned vigorous activity estimated using the sum of time spent in activities with MET values ≥ 6; Light-moderate activity was estimated using the sum of time spent in activities with MET values < 6

^f ^Occupational activities with a mean MET value of ≤ 1.5 (i.e. purely sedentary occupations) were excluded from activity estimates

### Agreement between EPIC questionnaire estimates and accelerometer readings

**Figure 2:**
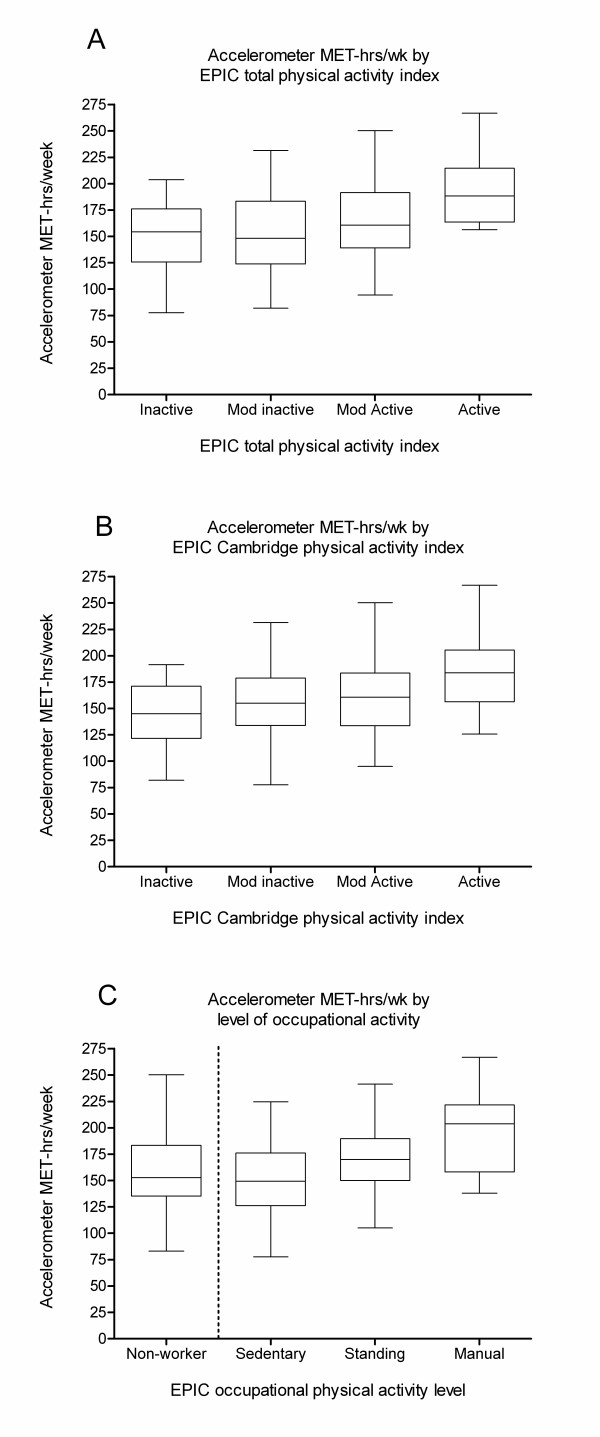
**Validity of the EPIC questionnaire physical activity categories compared to accelerometer measurement**. Box-and-whisker plots describing the validity of European Prospective Investigation into Cancer and Nutrition questionnaire physical activity categories compared to accelerometer measurement of total activity. The box represents the median and interquartile range, and the bars indicate the range. Graphs are presented for accelerometer MET-hours/week by: A. total physical activity index (Spearman rank correlation ρ = 0.29, 95% CI 0.15, 0.42), B. Cambridge physical activity index (0.32, 95% CI 0.19, 0.45), and C. occupational physical activity level (0.37, 95% CI 0.22, 0.51, excluding non-workers). *P *< 0.0001 for all measures.

Box-and-whisker plots (figure 2) demonstrate a positive association between accelerometer MET-hours/week and categorical measures of physical activity derived from the EPIC questionnaire, including the total physical activity index (ρ = 0.29, 95% CI 0.15, 0.42), the Cambridge index (0.32, 95% CI 0.19, 0.45) and occupational level index (0.37, 95% CI 0.22, 0.51). For each index, the most active group had noticeably higher median accelerometer MET-hours/week, but smaller differences were seen between the lower categories. Non-workers had similar levels of activity to participants in sedentary occupations. Similar results for these measures were seen when accelerometer-measured light activity was excluded, e.g. for the total physical activity index, ρ = 0.27 (95% CI 0.13, 0.40).

**Figure 3:**
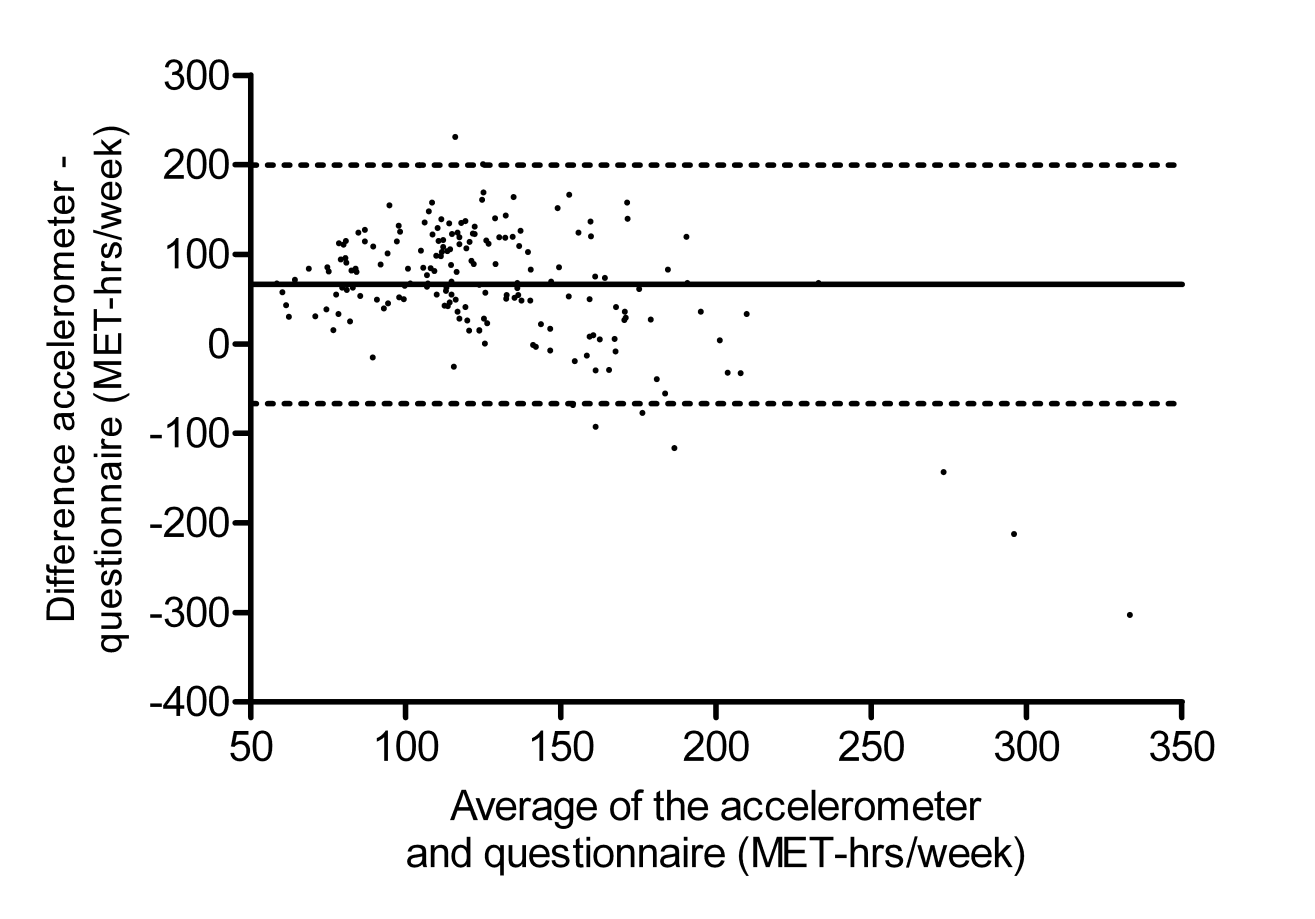
**Bland-Altman plot of EPIC non-occupational physical activity and total activity assessed by accelerometer measurement**. Bland-Altman plot of total non-occupational physical activity assessed by the European Prospective Investigation into Cancer and Nutrition questionnaire and total activity assessed by accelerometer measurement (both as MET-hrs/week). Mean difference: 66.4, standard deviation: 67.9, 95% limits of agreement: -66.7 to 199.6.

Table 3 shows that non-occupational (i.e. combined household and recreational) activity MET-hours/week assessed by the EPIC questionnaire were positively and significantly correlated (ρ = 0.21) with accelerometer measurements. A Bland-Altman plot of these data (figure 3) shows overall higher readings from the accelerometer than the EPIC questionnaire, but considerable variation in the individual differences between the EPIC and accelerometer estimates. As the level of physical activity (x-axis) increased, the mean difference between the accelerometer and EPIC measures (y-axis) decreased (correlation -0.28) but there also appeared to be more variation in the individual differences. The correlations appeared stronger for participants who were male, had a lower BMI, were younger, or were not full-time workers (table 3). However, none of these differences in correlations between subgroups were statistically significant (*P*_diff _>0.10), and we had limited power to detect these differences (<30% power for most inter-method *subgroup *comparisons in this study). The correlation for vigorous-intensity activity was 0.23 when MET-assigned intensities were used, and 0.18 when using self-rated intensity (table 3).

**Table 3:** Estimates of validity of non-occupational continuous physical activity measurements derived from the EPIC questionnaire when compared to accelerometer measurements of total physical activity

Accelerometer versus 10-month EPIC	MET-hours/week		
	
	Correlation (ρ)	95% CI	
Total non-occupational activity^a^			
Overall^b ^(n = 182)	0.21	0.07, 0.35	**
Gender			
Males (n = 100)	0.24	0.05, 0.42	*
Females (n = 82)	0.16	-0.06, 0.36	
Body mass index			
< 27.2 (n = 89)	0.33	0.14, 0.51	**
≥ 27.2 (n = 92)	0.12	-0.09, 0.32	
Age			
< 58 years (n = 95)	0.25	0.05, 0.43	*
≥ 58 years (n = 87)	0.18	-0.03, 0.37	
Employment status			
Full-time work (n = 113)	0.17	-0.02, 0.34	
Other (n = 68)	0.30	0.07, 0.50	*
Vigorous activity, self-rated^c^	0.18	0.04, 0.32	*
Vigorous activity, MET-assigned^c^	0.23	0.09, 0.37	**
Light-moderate activity^d^	0.19	0.05, 0.33	**

* *P *< 0.05; ** *P *< 0.01; *** *P *< 0.001

EPIC, European Prospective Investigation into Cancer and Nutrition; ρ, Spearman rank correlation coefficient; CI, confidence interval

^a ^EPIC total non-occupational activity = recreational + household physical activity; compared to total accelerometer activity (sum of light, moderate and vigorous activity)

^b ^There were no statistically significant differences in correlations for non-occupational activity between subgroups (*P*_diff _>0.10)

^c ^Time spent in vigorous non-occupational activity was measured (1) in a separate question about time in activities causing sweating or faster heartbeat, and (2), using the sum of time spent in activities with MET values ≥ 6 (i.e. cycling, sports and stair climbing); these estimates were compared to vigorous-intensity accelerometer activity.

^d ^Time spent in light-moderate non-occupational activity was estimated using the sum of time spent in activities with MET values < 6 (i.e. housework, walking, gardening, home repair); these estimates were compared to light+moderate-intensity accelerometer activity.

### Agreement between EPIC and Friedenreich LTPAQ questionnaire estimates

The correlation for the agreement of MET-hours/week between the baseline EPIC and LTPAQ questionnaires was 0.26 for total non-occupational activity, 0.21 for recreational activity, 0.46 for household activity, 0.40 for vigorous activity and 0.26 for light-moderate activities (table 4). The correlation for non-occupational activity appeared stronger for older than younger participants, however the correlations did not differ significantly (*P *= 0.17). The correlation for non-occupational activity was slightly higher (0.34, 95% CI 0.21–0.46) when the average of both EPIC questionnaires was compared to the LTPAQ.

**Table 4:** Comparison of non-occupational physical activity between the baseline EPIC and LTPAQ questionnaires

Baseline EPIC versus LTPAQ	MET-hours/week		
	
	Correlation (ρ)	95% CI	
Total non-occupational activity^a^			
Overall^b ^(n = 182)	0.26	0.11, 0.39	***
Gender			
Males (n = 100)	0.16	-0.04, 0.35	
Females (n = 82)	0.25	0.03, 0.44	*
Body mass index			
< 27.2 (n = 89)	0.27	0.07, 0.45	**
≥ 27.2 (n = 92)	0.22	0.02, 0.41	*
Age			
< 58 years (n = 95)	0.17	-0.03, 0.36	
≥ 58 years (n = 87)	0.36	0.16, 0.53	***
Employment status			
Full-time work (n = 113)	0.17	-0.01, 0.35	
Other (n = 68)	0.21	-0.03, 0.43	
Recreational activity	0.21	0.07, 0.34	*
Household activity	0.46	0.34, 0.57	***
Vigorous activity^c^	0.40	0.27, 0.52	***
Light-moderate activity^d^	0.26	0.12, 0.39	***

* *P *< 0.05; ** *P *< 0.01; *** *P *< 0.001

EPIC, European Prospective Investigation into Cancer and Nutrition; LTPAQ, Lifetime Total Physical Activity Questionnaire; ρ, Spearman rank correlation coefficient; CI, confidence interval

^a ^Total non-occupational activity = recreational + household physical activity

^b ^There were no statistically significant differences in correlations for non-occupational activity between subgroups (*P*_diff _>0.10)

^c ^Vigorous activity was estimated using the sum of time spent in activities with MET values ≥ 6 from each questionnaire

^d ^Light-moderate activity was estimated using the sum of time spent in activities with MET values < 6 from each questionnaire

### Long-term repeatability of EPIC questionnaire estimates

The repeatability of the EPIC physical activity questionnaire over 10 months is shown in table 5. The overall mean difference between the two administrations of the questionnaire was less than one MET-hour/week for total non-occupational and recreational activity, and 1.3 MET-hours/week for household activity. The Spearman correlation was 0.65 for total non-occupational activity, and was stronger for household (0.73) than for recreational activity (0.58) (*P*_diff _= 0.01). Light-moderate activities and vigorous activities had similar test-retest correlations. The weighted kappa coefficients were similar for the 'total' and 'Cambridge' index (0.62 and 0.66, respectively). Eighty-four percent of participants reported the same level of occupational activity on both administrations. The test-retest correlations were generally similar according to sex, BMI, age and employment status, although for household activity the correlation was higher for older (0.85, 95% CI 0.78, 0.90) than younger (0.59, 95% CI 0.45, 0.71) participants (*P*_diff _<0.001). Although the mean difference in MET-hours/week of non-occupational activity was small, a Bland-Altman plot of these data (not presented) showed wide 95% limits of agreement (-116.2, 115.0). A similar pattern was seen for recreational activity, and to a lesser extent, household activity (data not shown). When individual activities were examined, the repeatability was highest for housework (ρ = 0.77), followed by gardening (ρ = 0.73), sports and stair-climbing (both ρ = 0.69), cycling (ρ = 0.65), do-it-yourself activity (ρ = 0.47) and walking (ρ = 0.41).

**Table 5:** Repeatability of the EPIC physical activity questionnaire over 10 months

EPIC questionnaire measure	Mean (SD) difference^a^	Correlation (ρ)	95% CI
*Continuous measure (MET hours/week)*			
Total non-occupational activity	-0.6 (59.0)	0.65	0.55, 0.72
Recreational activity	0.7 (47.7)	0.58	0.48, 0.67
Household activity	-1.3 (33.5)	0.73	0.66, 0.79
Vigorous activity, self-rated^b^	3.3 (34.7)	0.63	0.54, 0.72
Vigorous activity, MET-assigned^b^	1.5 (32.4)	0.71	0.63, 0.78
Light-moderate activity^c^	-2.15 (51.0)	0.67	0.58, 0.74
			
*Categorical measure*		*K*_w_	
Total physical activity index		0.62	0.53, 0.71
Cambridge physical activity index		0.66	0.58, 0.74

*P *< 0.0001 for all measures.

EPIC, European Prospective Investigation into Cancer and Nutrition; ρ, Spearman rank correlation coefficient; CI, confidence interval; *K*_w _weighted kappa

^a ^Mean (SD) difference in MET-hours/week: EPIC 10-month minus baseline questionnaire.

^b ^Vigorous activity was estimated (1) in a separate question about time in activities causing sweating or faster heartbeat, (2) using the sum of time spent in activities with MET values ≥ 6

^c ^Light-moderate activity was estimated using the sum of time spent in activities with MET values < 6

## Discussion

The primary consideration of physical activity assessment in cancer epidemiological studies is to ensure appropriate and consistent categorisation of participants according to their total physical activity level. Our findings suggest fair agreement between the EPIC questionnaire and accelerometer measurements in the ranking of physical activity level, and satisfactory long-term repeatability of the EPIC questionnaire over a 10-month interval.

Estimates of total physical activity, encompassing occupational, recreational and household activity from the EPIC questionnaire, are based on a four-level 'total physical activity index'. This index was positively correlated (ρ = 0.29) with increasing accelerometer measurements, suggesting that the index is suitable for ranking overall level of total physical activity. However, in our study, the index appeared better at distinguishing the most active participants than those in the lower activity categories. The more active participants may have clearly defined patterns of physical activity that are more easily recalled, which could partly explain the greater agreement. There were similar positive associations for both the Cambridge index (ρ = 0.32) and the occupational level classification (ρ = 0.37). For each of these three indices there was a clear distinction between the most active and the least active group. Previously, studies have used heart rate monitoring [9], diaries [7] or indirect tests of validity based on predicted energy requirements [9,17], to assess the validity of the EPIC questionnaire. Heart rate monitoring was found to be associated with the Cambridge index but not with total activity that included lower-intensity activities [9]. Using accelerometry as an objective measure, we showed that the EPIC questionnaire does distinguish levels of total physical activity.

However, although the EPIC instrument can suitably rank participants according to physical activity level, there remains a considerable level of measurement error when assessing an individual's physical activity. Physical activity questionnaires with similar measurement characteristics to the EPIC questionnaire have been shown to lead to substantial attenuation of relative risk estimates for associations between physical activity and an outcome of interest, assuming the measurement errors are non-differential [29]. For example, an attenuation factor of 0.13 was estimated for the past-year total physical activity questionnaire developed by Friedenreich and colleagues, which translates to observing a relative risk of 1.10 instead of a true relative risk of 2.00 [29]. The correlation of 0.26 between the physical activity questionnaire and accelerometer measurement in their study [30] was similar to our results for the EPIC questionnaire, suggesting that a similar degree of attenuation may also be present when using the EPIC physical activity questionnaire to examine associations with disease outcomes.

The correlation between non-occupational physical activity from the EPIC questionnaire and accelerometer measurement was 0.21 overall, suggesting weak to fair agreement. It may, however, underestimate the true level of agreement because the accelerometer measurements include occupational physical activity, which is not necessarily well correlated with non-occupational activity since people who are physically sedentary at work might compensate by doing more recreational activity or vice-versa. In this population, for example, the correlation between these two components from the LTPAQ questionnaire was -0.12 (95% CI -0.27, 0.02). Thus, non-occupational physical activity is likely to be a better measure of total activity among those who are not employed, which is supported by our stratified results (ρ = 0.17 for full-time workers, ρ = 0.30 for non-workers/casual-workers). Despite this probable underestimation, the correlation of EPIC with accelerometer measurements is within the range of validity coefficients that have been shown with other self-report measures of adults' habitual or global physical activity: generally 0.14 to 0.36 [24,30,31]. Although we had limited power to evaluate the correlations between the accelerometer and EPIC measures among different subgroups, our results suggest that the EPIC questionnaire may be more accurate at ranking non-occupational physical activity levels among participants who were male, had a lower BMI, were younger, or were not full-time workers, which is consistent with other recent research [29,30].

The weighted kappa coefficients for the repeatability of the total physical activity index (0.62) and the Cambridge index (0.66) indicate good agreement [32] in classification of physical activity over a 10-month period. Wareham *et al *[9] reported slightly lower repeatability for the Cambridge index (0.60) over 18–21 months. The overall test re-test correlation of 0.65 for the continuous measure of non-occupational activity also indicates satisfactory long-term repeatability. Other questionnaires assessing past-year activity have shown similar estimates of repeatability [30,31]. Using a longer instrument interspersed with the EPIC physical activity questions, Pols *et al *[7] observed test re-test correlations over 5–11 months ranging from 0.47 to 0.89. The repeatability of the EPIC questionnaire may be underestimated in our study because of the long (10-month) interval between the first and second administration. Differences in self-reported activity between the repeat measures may thus reflect true changes in physical activity levels during the year in addition to recall error.

Measures of total and vigorous physical activity generally have higher repeatability coefficients than light-moderate intensity activities because they are usually more easily recalled [31,33-35]. However, we observed a higher repeatability correlation for household activity (0.73) than for recreational activity (0.58). Some previous studies have shown that light-moderate intensity household activities that are well-defined and routinely-performed, such as laundry, cooking, washing dishes or gardening, are easier to recall and have better measurement characteristics compared to more variable activities of similar intensity, such as walking [7,14,33,34,36,37].

Long-term exposures are thought to be more important than recent exposures in the aetiology of most cancers. Our data comparing the EPIC and Friedenreich LTPAQ questionnaires suggest that recent physical activity partially reflects lifetime activity, as recalled by the participants. The correlations were significantly higher for household activity than for recreational activity (0.46 vs. 0.21 respectively, *P*_diff _= 0.008), which may reflect the more variable nature of recreational activities throughout life compared to household activities that are regularly performed [14]. The slightly higher correlation (0.34 vs. 0.26) that was observed when we used the average of the two EPIC administrations (baseline and follow-up) suggests that repeat administration of the EPIC questionnaire would reduce intra-individual variation in physical activity [5]. Lack of strong agreement between the EPIC and LTPAQ questionnaires may reflect true differences between past-year and lifetime activity, in addition to different modes of administration. The LTPAQ has been shown to have high repeatability [14] but it is also a self-reported measure and may have similar measurement errors as those of the EPIC questionnaire.

Participants in our study are comparable to the EPIC cohort with regards to age, employment status and BMI [6,38,39], but the overall level of self-reported non-occupational activity was slightly higher in our study population [18]. Other factors, such as environmental and cultural differences between Europe and Australia, may affect the generalisability of our results to the EPIC cohort.

Our study has several strengths, including a large sample size, a fairly representative population, a high retention rate during follow-up, and the use of an objective validation measure that overcomes many of the inherent limitations in self-report methods [31]. Accelerometry is a valid and widely-used measure of total physical activity in adults [20,40], and unlike heart-rate monitoring, is able to detect low-moderate intensity activities. We used the Actigraph accelerometer, which has been shown to have little variability across individual units, and high overall reliability [21]. Three 7-day accelerometer monitoring periods were used during the 10-month study period, to ensure an accurate assessment of usual physical activity during the reference period, and to capture seasonal variations in physical activity. When comparing total MET-hours/week obtained from the first, second and third weeks of accelerometer measurement, the correlations were all in the range of 0.72–0.74, suggesting relatively little intra-individual variability in physical activity levels during the study period. Previous research has demonstrated that three to five days of accelerometer monitoring is sufficient to estimate habitual physical activity in adults reliably [41].

However, accelerometers are not a perfect gold standard measure of physical activity. Accelerometers alone cannot provide contextual information about the type or purpose of specific activities (e.g. work versus recreational activity), and they are limited in their ability to monitor upper body movements, water activities and movements with a weak vertical component such as cycling [40,42,43]. They may also underestimate some household-based activities involving upper body movements [43]. The choice of prediction equation and cut-points to categorise accelerometer time in different intensity categories may also influence results, although there is no optimal equation [40]. We chose the Swartz method [23] because it was derived using a broad age-group and range of field activities that best reflected our study population. We also focused on 'total' activity rather than intensity-specific activity.

For future use in epidemiological studies, some minor changes could be incorporated into the EPIC questionnaire that may improve its measurement characteristics and distinguish better between people who are sedentary or moderately inactive. Suggested improvements include i) capturing the frequency and duration of occupational activity, ii) changing the question on vigorous activity to mention 'breathing much harder than normal' rather than focus on 'sweating' (which can be weather dependent), and iii) splitting the housework activities into two or more categories (e.g. active childcare, cooking, cleaning), to assist with recall and to allow more precise estimation of intensity levels.

## Conclusion

Our findings suggest that the EPIC questionnaire has acceptable measurement characteristics for ranking participants according to their level of total physical activity. The EPIC questionnaire should be able to identify the presence or absence of reasonably strong aetiological associations when either recent or long-term activity is the responsible factor.

## Abbreviations

EPIC: European Prospective Investigation into Cancer and Nutrition; BMI: body mass index; CI: confidence interval; MET: metabolic equivalent values; ρ: Spearman rank correlation coefficient; SD: standard deviation; LTPAQ: Lifetime Physical Activity Questionnaire.

## Competing interests

The authors declare that they have no competing interests.

## Authors' contributions

AEC was the primary author responsible for design, statistical analysis, interpretation of data, and drafting and revising the manuscript, BJS, BKA, and AB helped conceive the study and formulate the study design, BJS, JC, HPV, CMF, BKA, and AB contributed to the interpretation of the data and critically revised the manuscript for important intellectual content, AEC, BJS, and JC contributed to collection of data. All authors read and approved the manuscript.

## Supplementary Material

Additional file 1 The EPIC physical activity questionnaire. Click here for file

Additional file 2 The total physical activity index, based on the cross-classification of occupational activity with combined recreational and household activity. Click here for file

Additional file 3 The classification of physical activity according to the Cambridge Physical Activity Index. Click here for file
