# Supplementary material for: Validity and repeatability of the EPIC physical activity questionnaire: a validation study using accelerometers as an objective measure
Source: Int J Behav Nutr Phys Act. 2008 Jun 2;5:33. doi: 10.1186/1479-5868-5-33 (PMC2424075; doi:10.1186/1479-5868-5-33)
Supplement: Additional file 1 — The EPIC physical activity questionnaire. [file 1479-5868-5-33-S1.doc]

**Additional file 1:** The EPIC physical activity questionnaire

1. Work

We would like to know the type and amount of physical activity involved in your work. Please check what the best corresponds with your present occupation from the following four possibilities:

- Sedentary occupation ____

You spend most of your time sitting (such as in an office)

- Standing occupation ____

You spend most of your time standing and walking. However, your work does not require intense physical effort (e.g. shop assistant, hairdresser, guard, etc.)

- Manual work ____

This involves some physical effort including handling of heavy objects and use of tools (e.g. plumber, electrician, carpenter, etc.)

- Heavy manual work ____

This implies very vigorous physical activity including handling of very heavy objects (e.g. docker, miner, bricklayer, construction worker, etc.)

1. In a typical week during the past year, how many hours did you spend per week on each of the following activities:

- walking, including walking to work, shopping and leisure time

in summer ____ hours per week

in winter ____ hours per week

- cycling, including cycling to work, shopping and leisure time

in summer ____ hours per week

in winter ____ hours per week

- gardening

in summer ____ hours per week

in winter ____ hours per week

- do-it-yourself activities at home

____ hours per week

- physical exercise such as fitness, aerobics, swimming, jogging, tennis, etc.

in summer ____ hours per week

in winter ____ hours per week

- housework, such as cleaning, washing, cooking, child care, etc.

____ hours per week

1. In a typical week during the past year, did you engage in any of these activities vigorously enough to cause sweating or faster heartbeat?

No ___ Yes ___

If yes, for how many hours per week in total did you perform vigorous activity?

____ hours per week

1. In a typical week during the past year, how many flights of stairs did you climb per day?

___ floors per day

**Note:**

Conversion of flights of stairs into a MET-hour/week variable:

20 steps/flight * 1min/72 steps * 1hr/60 min * #flights/day * 8 METS * 7 days/week

where the number of flights per day climbed is reported by the study subject and an assumption is made that the average number of steps climbed per minute is 72 and the average number of steps per flight is 20 (based on previous work).
