# Supplementary material for: Validity and repeatability of the EPIC physical activity questionnaire: a validation study using accelerometers as an objective measure
Source: Int J Behav Nutr Phys Act. 2008 Jun 2;5:33. doi: 10.1186/1479-5868-5-33 (PMC2424075; doi:10.1186/1479-5868-5-33)
Supplement: Additional file 2 — The total physical activity index, based on the cross-classification of occupational activity with combined recreational and household activity. [file 1479-5868-5-33-S2.doc]

**Additional file 2:** The total physical activity index, based on the cross-classification of occupational activity with combined recreational and household activity

| **Occupational Activity** | **Recreational and Household Activity**  (MET-hours/week in sex-specific quartiles) | | | |
| --- | --- | --- | --- | --- |
|  | Quartile 1 | Quartile 2 | Quartile 3 | Quartile 4 |
|  | Males: <50.0  Females: <56.7 | 50.0-66.0  56.7-91.1 | 66.1-97.0  91.2-155.6 | ≥97.1  ≥156.7 |
| Sedentary | Inactive | Inactive | Moderately inactive | Moderately active |
| Standing | Moderately inactive | Moderately inactive | Moderately active | Active |
| Manual | Moderately active | Moderately active | Active | Active |
| Heavy manual | Moderately active | Moderately active | Active | Active |
| Non-worker | Moderately inactive | Moderately inactive | Moderately active | Moderately active |
