# Supplementary material for: Validity and repeatability of the EPIC physical activity questionnaire: a validation study using accelerometers as an objective measure
Source: Int J Behav Nutr Phys Act. 2008 Jun 2;5:33. doi: 10.1186/1479-5868-5-33 (PMC2424075; doi:10.1186/1479-5868-5-33)
Supplement: Additional file 3 — The classification of physical activity according to the Cambridge Physical Activity Index. [file 1479-5868-5-33-S3.doc]

**Additional file 3:** The classification of physical activity according to the Cambridge Physical Activity Index

| **Work activity** | **Leisure time physical activity**  **(Duration of sport and cycling in hours/week)** | | | |
| --- | --- | --- | --- | --- |
|  | None | ≤ 3.5 | >3.5 and ≤ 7.0 | > 7.0 |
| Sedentary or non-worker | Inactive | Moderately inactive | Moderately active | Active |
| Standing | Moderately inactive | Moderately active | Active | Active |
| Manual | Moderately active | Active | Active | Active |
| Heavy manual | Active | Active | Active | Active |
